# αB Crystallin Is Apically Secreted within Exosomes by Polarized Human Retinal Pigment Epithelium and Provides Neuroprotection to Adjacent Cells

**DOI:** 10.1371/journal.pone.0012578

**Published:** 2010-10-08

**Authors:** Parameswaran G. Sreekumar, Ram Kannan, Mizuki Kitamura, Christine Spee, Ernesto Barron, Stephen J. Ryan, David R. Hinton

**Affiliations:** 1 Arnold and Mabel Beckman Macular Research Center, Doheny Eye Institute, Los Angeles, California, United States of America; 2 Department of Ophthalmology, Keck School of Medicine of the University of Southern California, Los Angeles, California, United States of America; 3 Department of Pathology, Keck School of Medicine of the University of Southern California, Los Angeles, California, United States of America; Roswell Park Cancer Institute, United States of America

## Abstract

αB Crystallin is a chaperone protein with anti-apoptotic and anti-inflammatory functions and has been identified as a biomarker in age-related macular degeneration. The purpose of this study was to determine whether αB crystallin is secreted from retinal pigment epithelial (RPE) cells, the mechanism of this secretory pathway and to determine whether extracellular αB crystallin can be taken up by adjacent retinal cells and provide protection from oxidant stress. We used human RPE cells to establish that αB crystallin is secreted by a non-classical pathway that involves exosomes. Evidence for the release of exosomes by RPE and localization of αB crystallin within the exosomes was achieved by immunoblot, immunofluorescence, and electron microscopic analyses. Inhibition of lipid rafts or exosomes significantly reduced αB crystallin secretion, while inhibitors of classic secretory pathways had no effect. In highly polarized RPE monolayers, αB crystallin was selectively secreted towards the apical, photoreceptor-facing side. In support, confocal microscopy established that αB crystallin was localized predominantly in the apical compartment of RPE monolayers, where it co-localized in part with exosomal marker CD63. Severe oxidative stress resulted in barrier breakdown and release of αB crystallin to the basolateral side. In normal mouse retinal sections, αB crystallin was identified in the interphotoreceptor matrix. An increased uptake of exogenous αB crystallin and protection from apoptosis by inhibition of caspase 3 and PARP activation were observed in stressed RPE cultures. αB Crystallin was taken up by photoreceptors in mouse retinal explants exposed to oxidative stress. These results demonstrate an important role for αB crystallin in maintaining and facilitating a neuroprotective outer retinal environment and may also explain the accumulation of αB crystallin in extracellular sub-RPE deposits in the stressed microenvironment in age-related macular degeneration. Thus evidence from our studies supports a neuroprotective role for αB crystallin in ocular diseases.

## Introduction

Age-related macular degeneration (AMD) is the most common cause of central vision loss in the elderly. The retinal pigment epithelium (RPE) is regarded as a primary site of pathology in AMD [1], [2]. The RPE forms a quiescent monolayer of non-proliferating cells, strategically located between the choriocapillaris/Bruch's membrane complex and the light-sensitive photoreceptors. The interphotoreceptor matrix (IPM) is a carbohydrate-rich complex that occupies the extracellular compartment between the outer neural retina and the apical surface of the RPE [3]. The IPM regulates the interaction between RPE and photoreceptors by exchanging nutrients, signaling molecules, and metabolic end products [4]. Although visual loss in early AMD is minimal, the RPE cells accumulate lipofuscin and are associated with the formation of extracellular deposits (drusen) in the macular region. Increasing numbers and size of macular drusen predispose to progression to the two late blinding forms of the disease. Advanced “dry” AMD is characterized by degeneration and loss of RPE with secondary loss of photoreceptors [5]. In contrast, advanced “wet” AMD is characterized by activation of RPE and growth of new, leaky vessels from the choroid. The vessels grow through breaks in Bruch's membrane to form a choroidal neovascular membrane adjacent to the RPE layer [6]. The RPE are therefore centrally involved in the pathogenesis of both late blinding forms of AMD.

The primary function of heat shock proteins (HSPs) is to prevent aggregation of folded proteins and to facilitate intracellular protein trafficking [7]. However, accumulating evidence suggests that HSPs are actively secreted and have important extracellular functions [8]. Given the critical intracellular roles played by HSPs, the existence of secretory pathways that allow cells to release HSPs, both under steady state and under stressed conditions, may appear counterintuitive. The most widely recognized mechanism for protein release from the cells is the classical pathway involving endoplasmic reticulum and Golgi. However, both non-classical and alternative pathways are also involved in protein secretion [9].

Like other HSPs, αB crystallin is a molecular chaperone that is induced by an array of stress stimuli and that offers cytoprotective effects by suppressing aggregation of proteins [10] and disrupting the proteolytic action of caspase 3 [11]. αB Crystallin is a mitochondrial and cytosolic protein [12], [13]. As the major α-crystallin in the RPE, αB crystallin provides significant protection against oxidative stress [12], [14]. αB Crystallin shows increased expression in the RPE in AMD, suggesting that it may represent a stress response to protect RPE in AMD, and it may be considered as a biomarker of the disease [15]. Interestingly, αB crystallin is also found in extracellular drusen deposits and has been reported as a component of the IPM, suggesting the possibility that it may be secreted [16], [17].

We hypothesized that exosomes mediate the release of αB crystallin from RPE cells and that, in polarized RPE monolayers, αB crystallin secretion is asymmetrical towards photoreceptors where it accumulates in the IPM. The extracellular αB crystallin is internalized into neighboring cells (photoreceptors and RPE) under stressed conditions, where it could protect the cells from oxidative injury. We also hypothesized that severe oxidative stress results in RPE cell death and the release of αB crystallin to the choroidal side, leading to its accumulation in drusen. To test these hypotheses, we performed mechanistic studies with RPE cells to establish that secretion is exosome dependent. Further, asymmetry in αB crystallin secretion in the presence or absence of oxidative stimulus was investigated using highly polarized human RPE monolayers. In other studies, uptake of exogenous αB crystallin both by RPE cells and mouse retinal explants and protection from cell death was examined under conditions of oxidative injury.

## Methods

### Ethics statement

This study conforms to applicable regulatory guidelines at the University of Southern California, principles of human research subject protection in the Declaration of Helsinki and principles of animal research in the Association for Research in Vision and Ophthalmology Statement for the Use of Animals in Ophthalmic and Vision Research. The Institutional Review Board (IRB) of the University of Southern California approved our use of human RPE cells under protocol #HS-947005 (continuing review approved June 2, 2010). The University of Southern California Institutional Animal Care and Use Committee approved our animal studies under protocol # 11135 (continuing review approved January 27, 2010).

### Secretion studies in non-polarized and polarized RPE cell cultures

Detailed description of isolation and culturing of human RPE and a protocol for generation of long-term polarized RPE cultures were given in our earlier publications [18], [19], [20]. Prior to experiments, cells were switched to exosome-free medium to avoid contamination from endogenous exosomes present in the serum.

For determining αB crystallin secretion from confluent non polarized RPE cells, the extracellular medium from one T75 flask containing 6–7×10^6^ human RPE cells was collected, centrifuged to remove dead cells and concentrated using centrifugal filter devices (10 kDa cutoff, Millipore, MA) to 20 µl for Western blot analysis and 100 µl for ELISA analysis. To examine the involvement of the classical secretory pathway, RPE cells were pretreated for 2 h with 7 µg/ml brefeldin (BF), and 5 µg/ml tunicamycin (TM) as described [21], [22], and 24 h secretion of αB crystallin was measured. To exclude the possibility of contamination of αB crystallin released from dead and lysed cells, cell viability was monitored in all experiments by assay of LDH activity (Clontech, CA).

Human RPE cells in long-term culture with transepithelial resistance (TER)>300 Ω·cm^2^ were used in experiments with polarized RPE cells. After 24 h, extracellular medium from the apical and basolateral sides was collected separately. Pooled media (4 ml from apical side and 4 ml from basal side) from 4 transwells with ∼700,000 cells/well at confluence was concentrated and analyzed as described above for non-polarized RPE. Recombinant human αB crystallin (rhαB crystallin) was used as a positive control in all Western blot analyses.

### Exosome extraction and characterization

Exosomes were isolated by differential centrifugation from conditioned media of RPE cells following a protocol described for cell culture supernatants [23]. Briefly, spent media was collected from 6–7×10^6^ cells after 24 h in serum free media. The harvested supernatant was subjected to differential centrifugation at 4°C, starting with a centrifugation at 300×g (10 min) and followed by centrifugations at 2000×g for (10 min), 10,000×g (30 min) and 100,000×g (70 min) as described [23]. The resulting exosome pellet was resuspended in 10 ml sterile PBS and centrifuged again at 100,000×g for 70 min. The pellet was resuspended in 50 µl 2% paraformaldehyde and processed for transmission electron microscopy (TEM) and immunogold labeling for αB crystallin or processed for Western blot analysis.

### Pharmacological inhibition of exosomes

Lipid rafts play a significant role in heat shock protein release. RPE cells were pretreated for 2 h with 25 µg/ml methyl-β-cyclodextrin (CD) or 25 µg/ml dimethyl amiloride (DMA) (Sigma, MO) [21], [22], [24]. In both cases, the amount of αB crystallin secreted into the medium in the presence of inhibitors was determined and compared to untreated controls.

### Localization of αB crystallin and CD63 in polarized RPE

RPE monolayers grown on transwell filters were fixed in ice-cold methanol followed by three washes in phosphate buffered saline (PBS) and subsequently permeabilized with 0.1% Triton-X 100 for 15 min. Specimens were blocked in 5% BSA before incubating with αB crystallin rabbit polyclonal antibody (1∶100 dilution, Stressgen, CA) and CD63 mouse monoclonal antibody (1∶100 dilution, Abnova, Taiwan) at 4°C overnight. The cells were washed and incubated with fluorescein and rhodamine conjugated anti-rabbit/anti-mouse secondary antibody (Vector Labs, CA) for 30 min at room temperature. After immunostaining, membranes were removed from the inserts with a fine, sharp, sterile razor by inserting it at one side of the filter and then gently moving it around the filter. The specimen was viewed on an LSM 510 laser-scanning microscope (Carl Zeiss, Thornwood, NY).

### ELISA assay for αB crystallin

Results obtained by Western Blot analysis were further confirmed by an optimized in-house enzyme-linked immunosorbent assay (ELISA). Briefly, microtiter plates were coated overnight with 2.5 µg/ml rabbit polyclonal αB crystallin capture antibody (Stressgen, MI). After blocking with 1% BSA, both standards and samples (100 µl) were added in duplicate and incubated for 1 h at room temperature. After 1 h incubation with detection antibody and subsequent rinsing, the assay was performed by incubation with tetramethylbenzidine substrate, and intensity was measured in a microplate reader (Bio-Rad, CA) at 450 nm. The interassay variations for αB crystallin averaged 7.5% and the assay was linear in the range of 1.25 to 40 ng/ml (r^2^ = 0.996). Extracellular or cellular αB crystallin concentrations were measured using the standard linear plot obtained from the optical density readings of recombinant αB crystallin standards.

### Localization of αB crystallin in the interphotoreceptor matrix

Retinal cryosections (8 mm) from 24 h dark adapted 129 svE mice were air-dried, fixed, and processed as described [14] using mouse monoclonal antibody against bovine αB crystallin (Stressgen, MI) and rabbit polyclonal antibody against an interphotoreceptor retinoid-binding protein (IRBP) (Santa Cruz Biotechnology, CA). Sections were viewed under a confocal microscope (Carl Zeiss, Thornwood, NY).

### Immunogold labeling of αB crystallin in the interphotoreceptor matrix

Retinal tissues were fixed in 2% paraformaldehyde and 0.1% glutaraldehyde in phosphate buffer (pH 7.4) for 1 h at room temperature. The fixed tissues were dehydrated and embedded in 100% LR White acrylic resin (Ted Pella Inc, CA). The blocks were then ultra thin sectioned (75 nm in thickness) and placed on parlodian coated nickel grids. Sections on grids were etched with 0.5% sodium metaperiodate, blocked in 5% BSA and incubated with rabbit polyclonal αB crystallin antibody (Stressgen, MI) at 1∶75 dilution overnight. The grids were rinsed in PBS, incubated in secondary antibody conjugated to 15 nm gold (Ted Pella Inc, CA.) and counterstained with saturated uranyl acetate. Grids were viewed under a digital electron microscope (JEOL-2100, USA) at 100 KV.

### Fluorescein labeling of recombinant human αB crystallin

Following the manufacturer's instructions, rhαB crystallin was labeled with Fluorescein Labeling Kit-NH2 (Dojindo Molecular Technologies, MD). In short, 100 µg of recombinant human (rh) αB crystallin protein was mixed with 100 µl wash solution, followed by centrifugation in a filtration tube. The NH2-reactive fluorescein and reaction buffer was added to the filtration tube, incubated 10 min at 37°C, followed by centrifugation and washing. The labeled protein retained in the filter device was washed in PBS and the ratio of fluorescein and IgG was determined by a spectrophotometer.

### Protection of RPE by exogenous αB crystallin from H_2_O_2_-induced cell death

The effect of co-treatment with αB crystallin was studied in confluent human RPE cells challenged with either 500 µM H_2_O_2_ alone or together with 25 µg/ml rhαB crystallin for 3 h. Cell death was quantitated by TUNEL assay and active caspase 3 [20]. The number of TUNEL-positive cells was counted under a fluorescent microscope and the average number of apoptotic cells was recorded [20].

### Uptake of labeled rhαB crystallin by mouse retinal explants

Mouse eyes (129 svE, 6 weeks old) were enucleated and incubated in culture medium (DMEM) supplemented with 1% FBS. The anterior segment, lens, and vitreous body were then removed. The whole retina with very little or no RPE was gently removed from the eye cup and flat-mounted in a 96 well tissue culture plate and incubated with either 500 µM H_2_O_2_ and 25 µg/ml fluorescein labeled rhαB crystallin or labeled rhαB crystallin alone. Uptake of labeled rhαB crystallin by the neural retina was examined at different time points (15 min, 30 min, 45 min and 60 min) under a confocal microscope.

### Data analysis

Data presented are mean ± SD. Statistical analysis was performed by one way analysis of variance, followed by post test using InStat software (GraphPad Software, Inc., San Diego, CA). A *p* value of less than 0.05 was considered statistically significant.

## Results

### αB Crystallin is secreted from human fetal RPE cells

**Figure 1:**
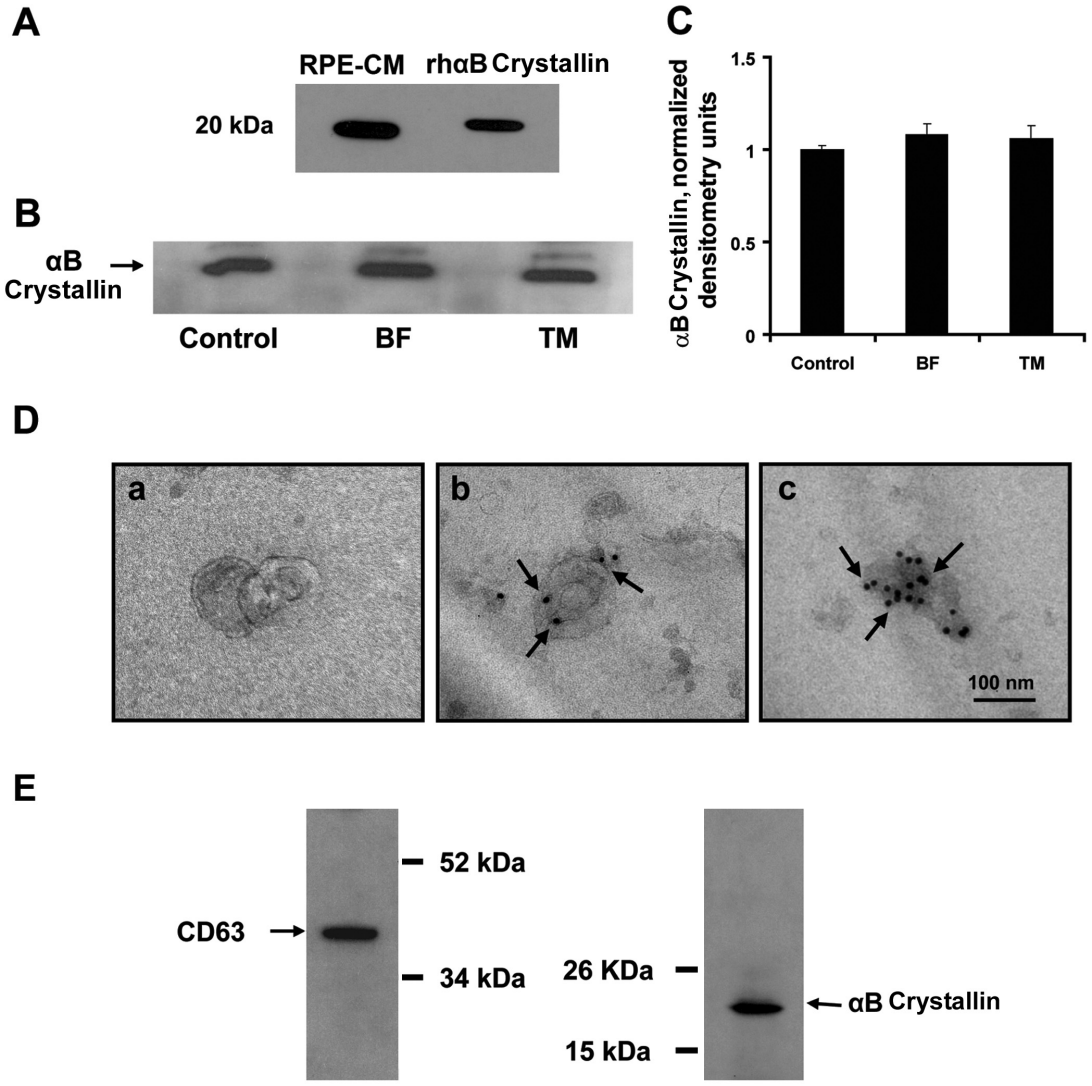
Secretion of αB crystallin from confluent human RPE cells. (A) Secretion was determined from extracellular RPE conditioned medium (RPE-CM) collected at 24 h by Western blot analysis using rhαB crystallin as a positive control. Extracellular release of αB crystallin is independent of classical protein transport pathways but is dependent on exosomes. (B,C) Effect of pretreatment of RPE with 7 µg/ml brefeldin (BF) and 5 µg/ml tunicamycin (TM) on secretion in 24 h. (D) Characterization and localization of αB crystallin in exosomes by TEM. Exosomes averaged 100 nm in size. (panel a, whole-mounted exosomes). Immunogold labeling of αB crystallin (15 nm gold particles) within exosomes (arrows, panel b,c) in independent preparations. Scale bar = 100 nm. (E) Expression of CD63 (left) under nonreducing conditions and αB crystallin (right) under reducing conditions in RPE exosomal fractions.

We used confluent, early passage human RPE cells (7×10^6^ cells/T75 Flask) to determine secretion. αB crystallin secreted to the medium in a 24 h period was measured by Western blot analysis (Figure 1A). Quantitation of secretion was achieved by an independent ELISA method which showed linearity in the range of 1.25–40 ng/ml of αB crystallin (Figure S1). Secretion from non-polarized confluent RPE was 1.08±0.10 ng/10^6^ cells in 24 h (Figure S1, inset) while αB crystallin levels in cytosol and mitochondria of RPE cells were much higher. Thus, secretion accounted for a portion of the steady state level found in cytosol and mitochondria.

### αB Crystallin secretion occurs independent of the classical secretory pathway

Treatment of RPE cells for 2 h with an inhibitor of ER-Golgi transport (brefeldin; BF) or N-linked glycosylation (tunicamycin; TM) resulted in no significant change in αB crystallin secretion (Figure 1B,C). Cell viability experiments to exclude the possibility of contamination from dead cells showed the mean percent cell viability of RPE after 2 h pretreatment with 7 µg/ml BF and 5 µg/ml TM were 95.2 and 94.5, respectively, vs. that of untreated controls (96.8). Lactate dehydrogenase (LDH) release under the experimental conditions showed no significant difference as compared to controls (Figure S2). Taken together, these data exclude the participation of the common secretory pathway for αB crystallin release from RPE cells.

### αB Crystallin is secreted via exosomes and secretion is lipid raft dependent

Next, the possibility that αB crystallin is secreted via exosomes was tested. Exosomes isolated from RPE cells maintained in serum free medium for 24 h were characterized by transmission electron microscopy (TEM) and Western blot analysis. Exosomal preparations derived from RPE cells exhibited morphology typical of exosomes (Figure 1D, Panel a). Immunogold labeling with αB crystallin showed immunogold particles within the exosomes suggesting an exosomal-dependent secretion pathway (see arrows, Figure 1D, Panels b, c show exosomes from 2 independent experiments). The exosomal marker protein CD63 was enriched in the fractions obtained (Figure 1E), and Western blot analysis showed that the exosomal fraction contained αB crystallin (Figure 1E).

**Figure 2:**
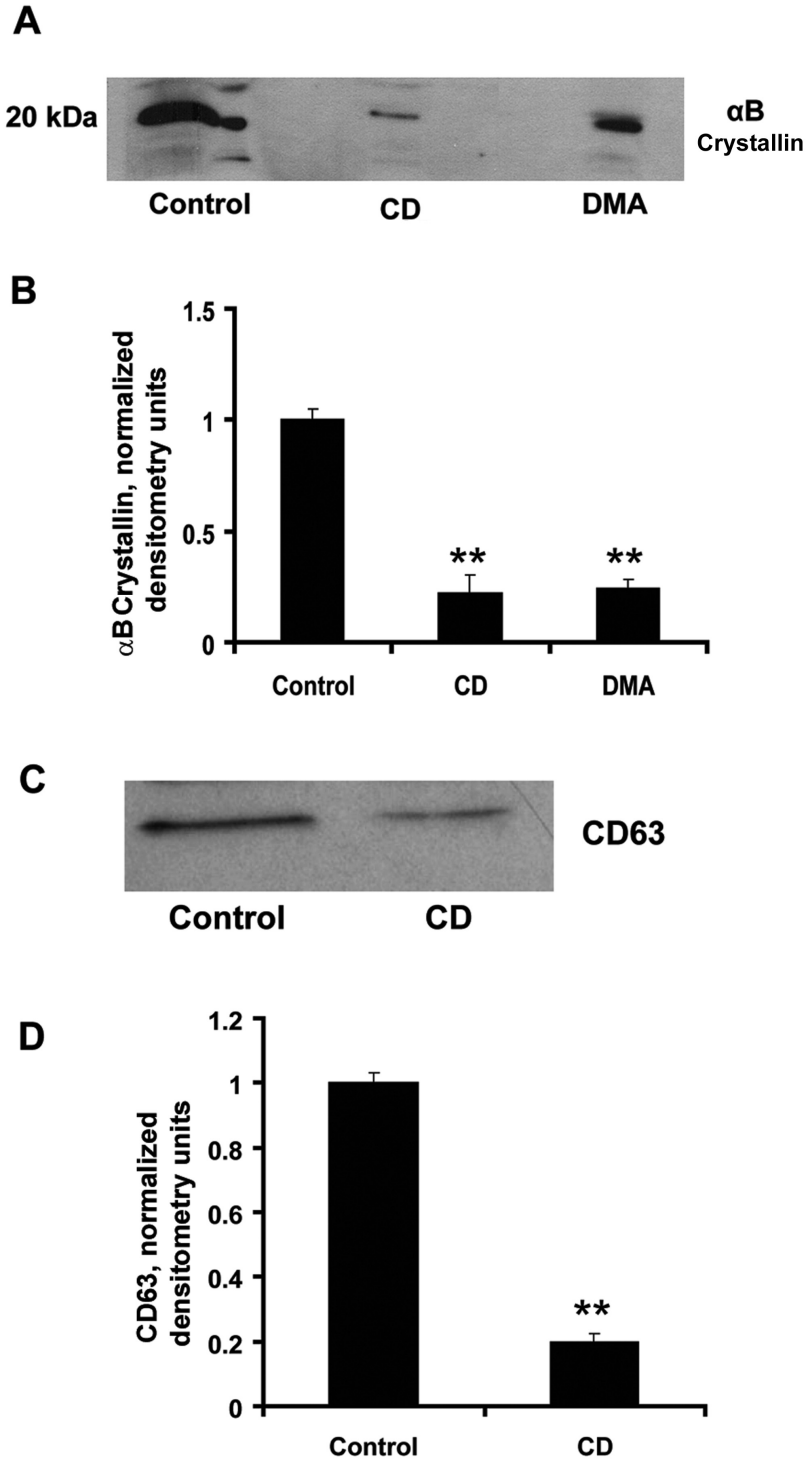
Exosome inhibitors significantly reduced αB crystallin release from RPE cells. (A,B) αB Crystallin secretion in 24 h was determined and quantified after 2 h exposure to 25 µg/ml of β-methyl cyclodextrin (CD) or dimethyl amiloride (DMA). (C,D) Western blot analysis and its quantification of CD63 showing inhibition of exosomes by treatment of RPE cells with 25 µg/ml CD. ** indicates *p*<0.01 vs controls from 4 determinations/group.

Known pharmacological inhibitors of exosomes were used to further confirm the phenomenon of exosomal-dependent secretion of αB crystallin [20], [23]. Our data revealed that 2 h pretreatment of RPE cells with 25 µg/ml of β-methyl cyclodextrin (CD, a lipid raft and cholesterol depletor) as well as 25 µg/ml dimethyl amiloride (DMA, exosome inhibitor and inhibitor of H^+^/Na^+^ and Na^+^/Ca^2+^ exchanger) significantly inhibited αB crystallin secretion (*p*<0.01 vs. controls, Figure 2A, B). DMA treatment caused a significant 80% decrease in exosome release as evidenced by a decrease in CD63 expression in exosomal fractions (Figure 2C,D). Cell viability remained >95% under all experimental conditions and LDH release was unaltered, indicating lack of membrane disruption (Figure S2).

### αB Crystallin is selectively secreted to the apical domain in polarized RPE cells

**Figure 3:**
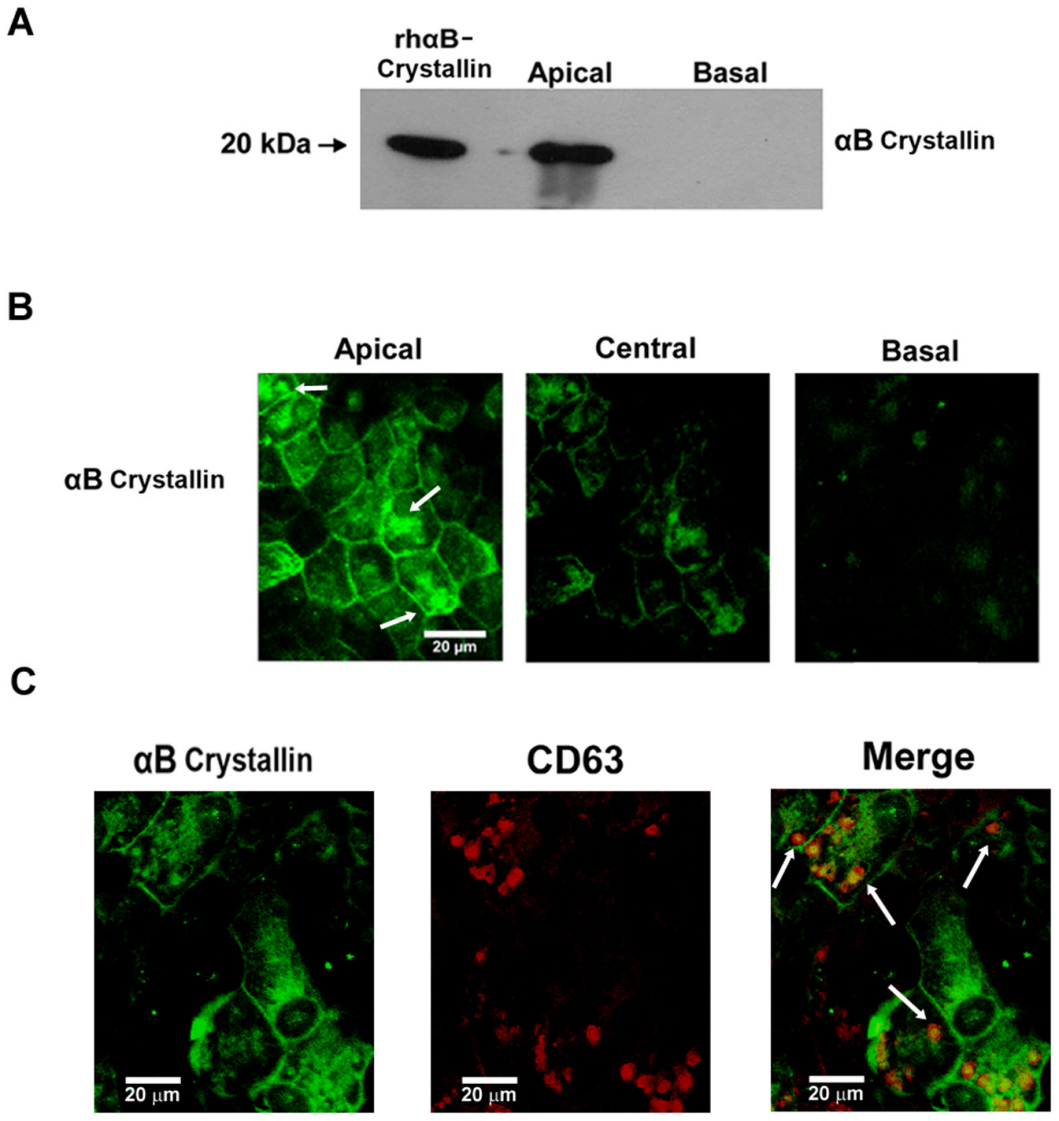
Preferential secretion of αB crystallin into the apical domain of polarized RPE monolayers. (A) Secretion of αB crystallin from human RPE monolayers (mean TER 386 Ω·cm^2^) was measured from apical and basolateral medium. (B) Immunofluorescence staining of αB crystallin in RPE monolayers showing predominant apical staining as compared to central and basal regions. (C) Co-localization of αB crystallin (green) with CD63 (red) in polarized RPE cells. Scale bar = 20 µm.

The asymmetry of secretion of αB crystallin was investigated in polarized human RPE monolayers. Polarized RPE monolayers were prepared [18], [19] and characterized by tight junction proteins occludin, ZO-1, and apical membrane marker Na/K ATPase (Figure S3). The average TER of the RPE cultures at the time of experimentation was 386±68 Ω·cm^2^. Under unstressed conditions αB crystallin was secreted to the apical side facing the photoreceptors with no detectable secretion to the basolateral side facing the choroid (Figure 3A). ELISA assay showed a secretion of 5.26 ng/10^6^ cells/24 h αB crystallin to the apical side while secretion to the basolateral side was below the detection limit. Cellular localization studies by confocal microscopy confirmed that αB crystallin is predominantly localized to the apical region of the cell (Figure 3B), supporting its observed apical secretion. The finding that αB crystallin secretion is exosome dependent led us to examine whether αB crystallin co-localized with exosomes. In RPE monolayers, αB crystallin was partially co-localized with CD63 (Figure 3C), strongly supporting the hypothesis that the release of αB crystallin is through the exosomal route.

### Barrier properties of RPE are compromised with severe oxidative stress and affect polarity of αB crystallin secretion

**Figure 4:**
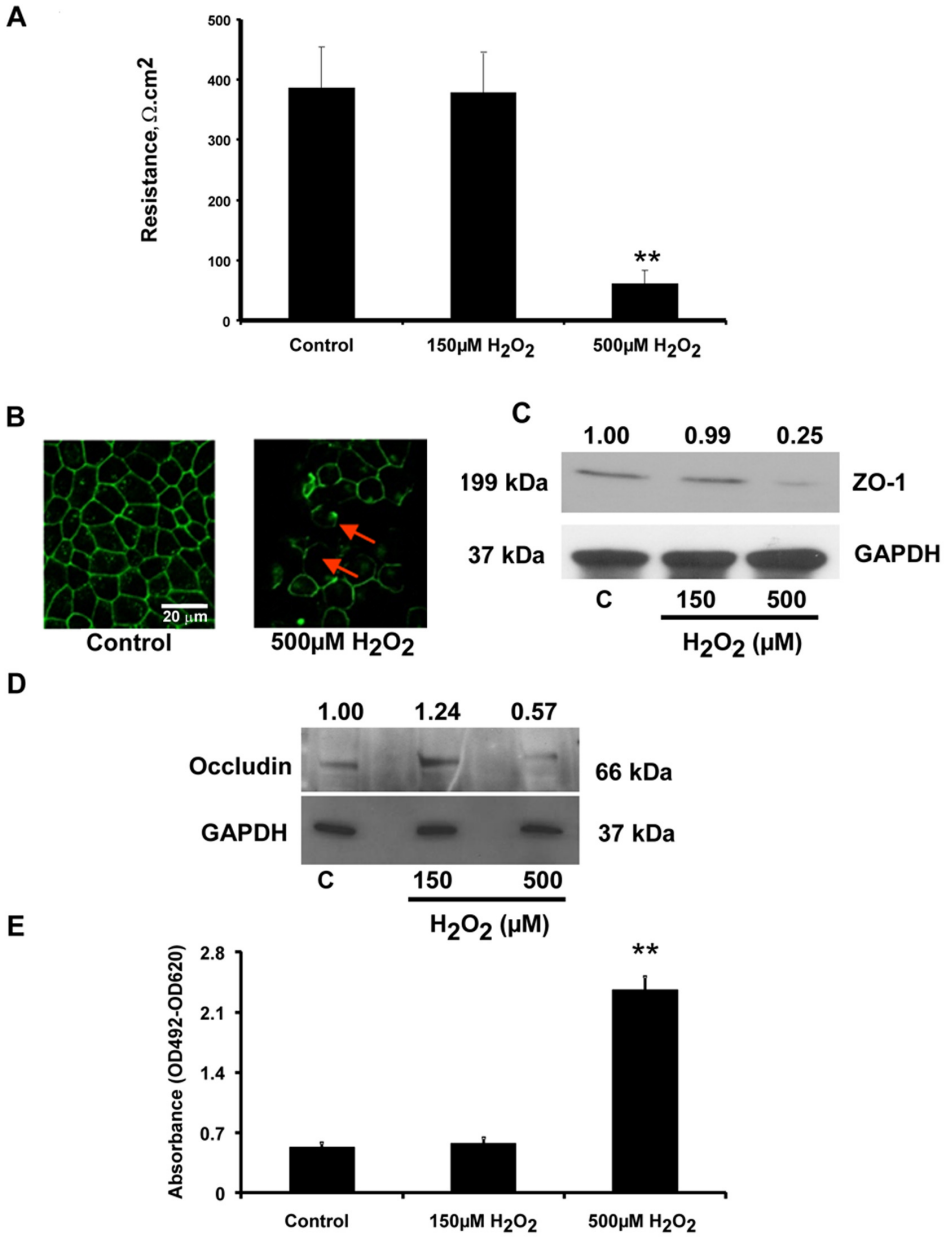
Effect of oxidative stress on functional properties of polarized RPE monolayers. (A) Severe oxidative stress significantly decreased TER of polarized RPE monolayers. Only a high dose of H_2_O_2_ (500 µM) produced a significant decrease in TER in 24 h vs untreated controls. (B, C) Confocal microscopy image showing breaks (red arrows) in ZO-1 staining with 500 µM H_2_O_2_, verified by Western blot analysis which showed a significant decrease in ZO-1 protein expression with 500 µM H_2_O_2_. (D) Western blot analysis of occludin showing a significant decrease with severe but not with mild H_2_O_2_ treatment. Values marked above the gels are from densitometry measurements. (E) LDH release measured as a marker for membrane disruption, was significantly higher (*p*<0.001 vs control) with severe oxidative stress, but was unchanged with mild stress. ** denotes *p*<0.01 vs control values. Scale bar = 20 µm in panel B.

**Figure 5:**
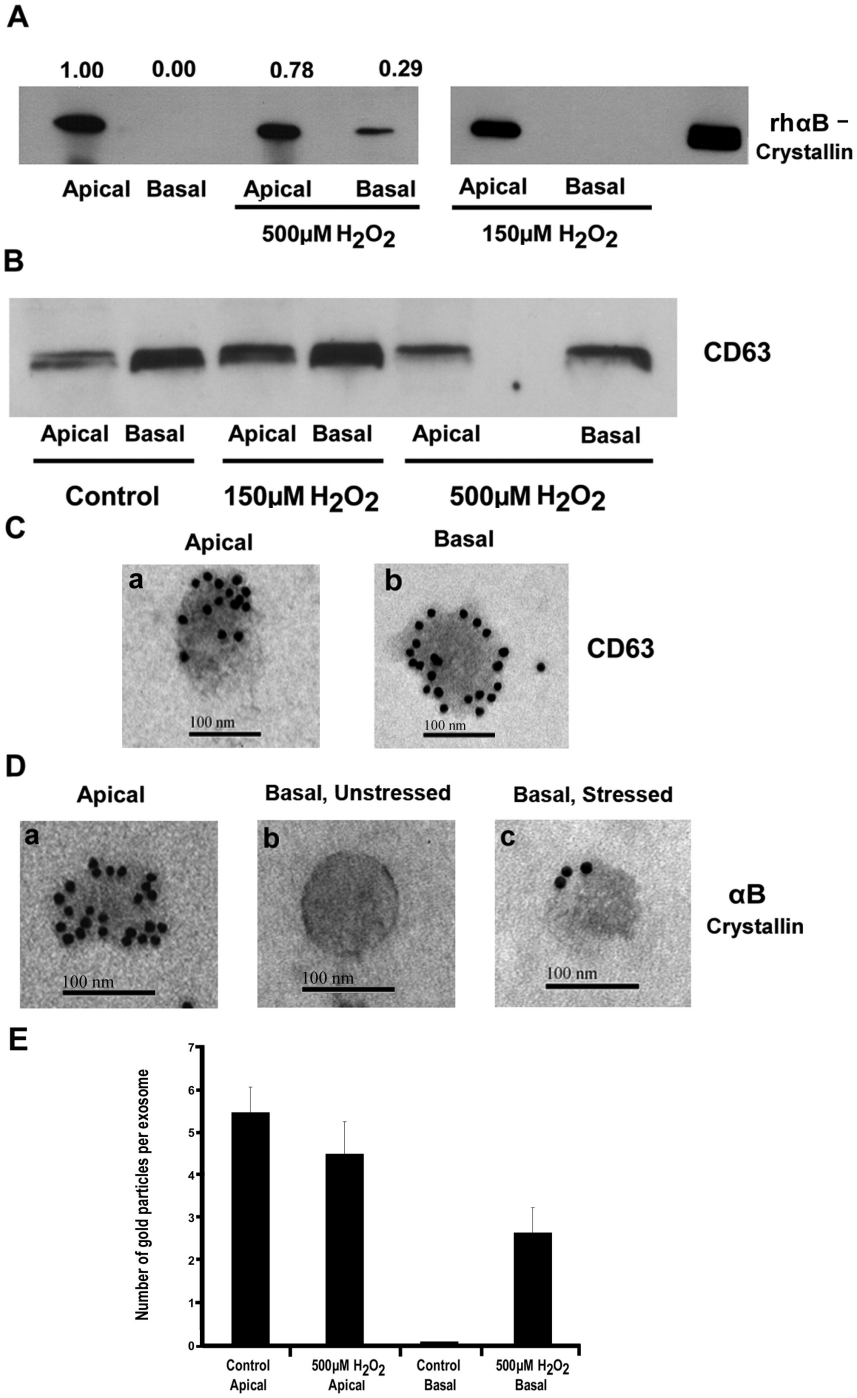
αB Crystallin secretion in polarized RPE cells under conditions of oxidative stress. (*A*) Polarized RPE cells were challenged with severe (500 µM H_2_O_2_-24 h) or mild (150 µM H_2_O_2_ - 24h) oxidative stress and αB crystallin secretion was determined. Values marked on the top of the blot are densitometric ratios, normalized to apical secretion of unstressed RPE taken as unity. (B) Protein expression of the exosomal marker CD63 in the medium of RPE treated with 150 µM and 500 µM H_2_O_2_. (C) Immunogold labeling of CD63 with 15 nm gold particles in the exosomes isolated from apical (Panel a) and basolateral (Panel b) medium. (D) Immunogold labeling of αB crystallin (15 nm gold particles) in the exosomes isolated from apical (panel a) and from basolateral medium (panel b, c). (E). Gold particles were counted from individual exosomes positive for αB crystallin and data are expressed as average number of gold particles per exosome (n = 61–100). No significant difference in gold particles/exosome was observed for exosomes isolated from apical media from unstressed and stressed cells. However, a significant (p<0.001) increase in αB crystallin localization in exosomes was observed in exosomes isolated from basal medium of RPE cells subjected to severe oxidative stress. Scale bar = 100 nm for C and D.

It has recently been proposed that increased autophagy and release of proteins through exosomes by the aged RPE may contribute to drusen formation [25]. Given the link between aging and oxidative stress, the effect of mild (150 µM H_2_O_2_ for 24 h) and severe (500 µM H_2_O_2_ for 24 h) oxidative injury on secretory properties of RPE monolayers was tested. Severe oxidative stress resulted in a significant (*p*<0.001 vs untreated controls) loss of TER (Figure 4A) caused by disruption in the pattern and loss of tight junction protein expression (Figure 4B,C,D) and significant cell death (*p*<0.001 vs controls) (Figure 4E). Further, unlike in the unstimulated RPE, secretion of αB crystallin also occurred at the basolateral side facing the choroid (Figure 5A). It must be noted, however, that a significant amount of secretion to the apical side was still present (Figure 5A). This phenomenon of partial switch to basolateral side occurred only when RPE was subjected to severe oxidative stress, while relatively mild oxidative stress did not affect the nature of polarized secretion (Fig 5A), tight junction protein expression or TER (see Figure 4A,B,C,D above). In separate experiments, we also confirmed that both the apical and basolateral sides of polarized RPE release exosomes (Figure 5B, C). These studies indicated that the absolute amount of exosomes in the basolateral domain was higher than that in the apical side (Figure 5B). αB Crystallin immunolabeling was seen in the exosomal fractions of the apical side in both the non-stressed and stressed states (Figure 5D, panel a, showing non-stressed). However, αB crystallin labeling was found only in the exosomes isolated from the basolateral compartment of severely stressed cells (Figure 5, panel c) and not in those isolated from unstressed cells (Figure 5, panel b). We quantified the average number of gold particles/exosome from the apical as well basal sides of stressed and non-stressed conditions (Figure 5E). A significant (*p*<0.001) increase in αB crystallin immunogold labeling was observed in exosomes isolated from basolateral media of severely stressed RPE cells.

### Localization of αB crystallin in the mouse retina

**Figure 6:**
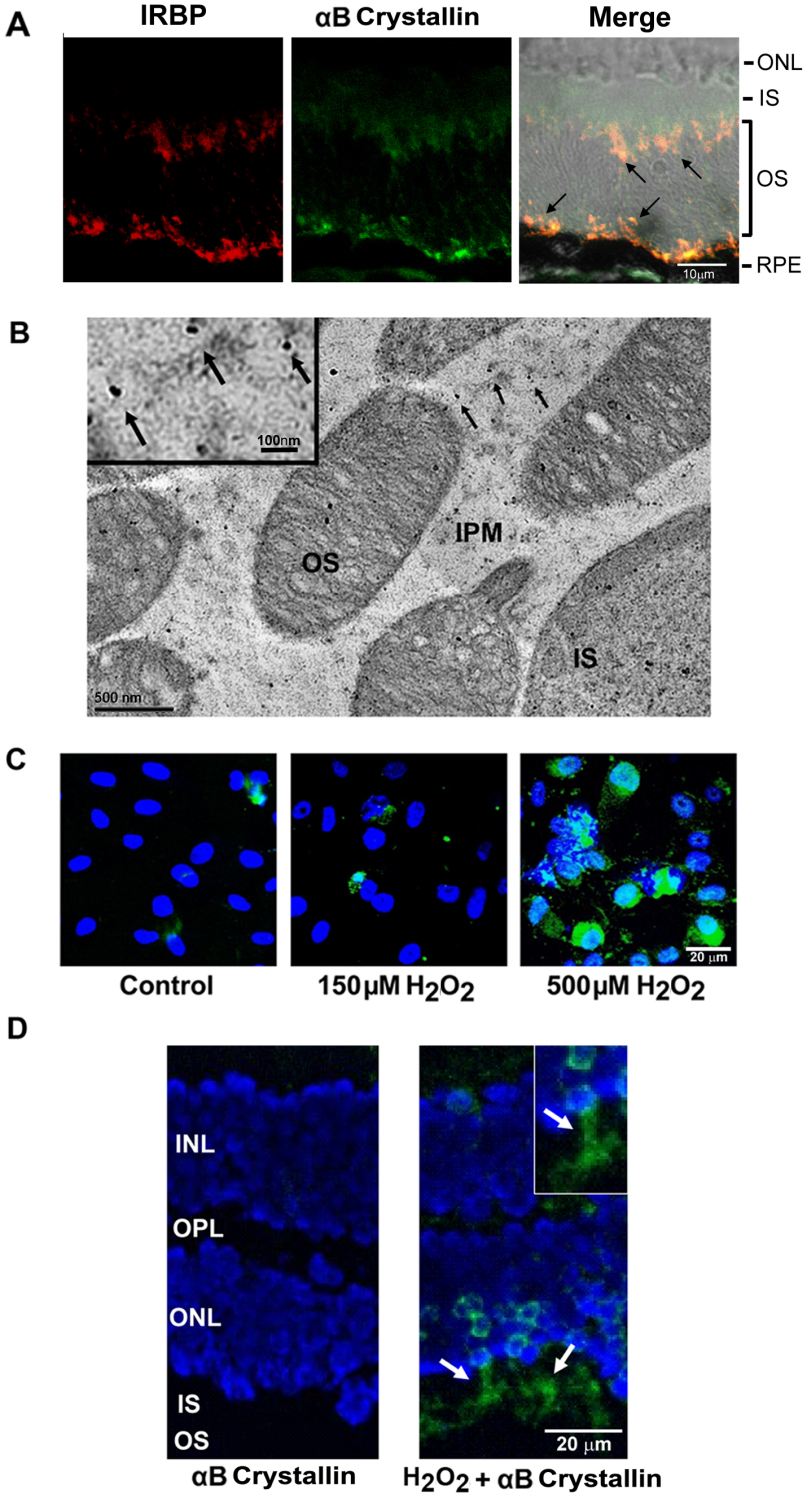
Extracellular localization of αB crystallin in murine IPM in retinal tissue sections, and uptake of exogenous αB crystallin in RPE and photoreceptors *in vitro*. (A) Double staining for interphotoreceptor retinoid-binding protein (IRBP, red) and αB crystallin (green) demonstrating partial co-localization. Arrows indicate yellow staining in the merged image for αB crystallin and IRBP in the IPM as determined by confocal microscopy. (B) TEM of murine retinal sections shows distribution of αB crystallin (15 nm gold particles, arrows in the figure and in inset) in the IPM and photoreceptor inner (IS) and outer segments (OS). (C) Uptake of fluorescein labeled rhαB crystallin in the presence or absence of oxidative stress (150 or 500 µM H_2_O_2_). Nuclear and cytoplasmic uptake of αB crystallin (green) is seen after 1 h treatment which is more pronounced with 500 µM H2O2. (D) Uptake of fluorescein labeled 25 µg/ml rhαB crystallin in photoreceptors of mouse retinal explant cultures co-treated with 500 µM H_2_O_2_ for 30 min (arrows in right panel and in inset). Unstressed control is shown in the left panel. The scale bars represent: A (10 µm), C, D (20 µm) and B (500 nm; 100 nm for inset).

**Figure 7:**
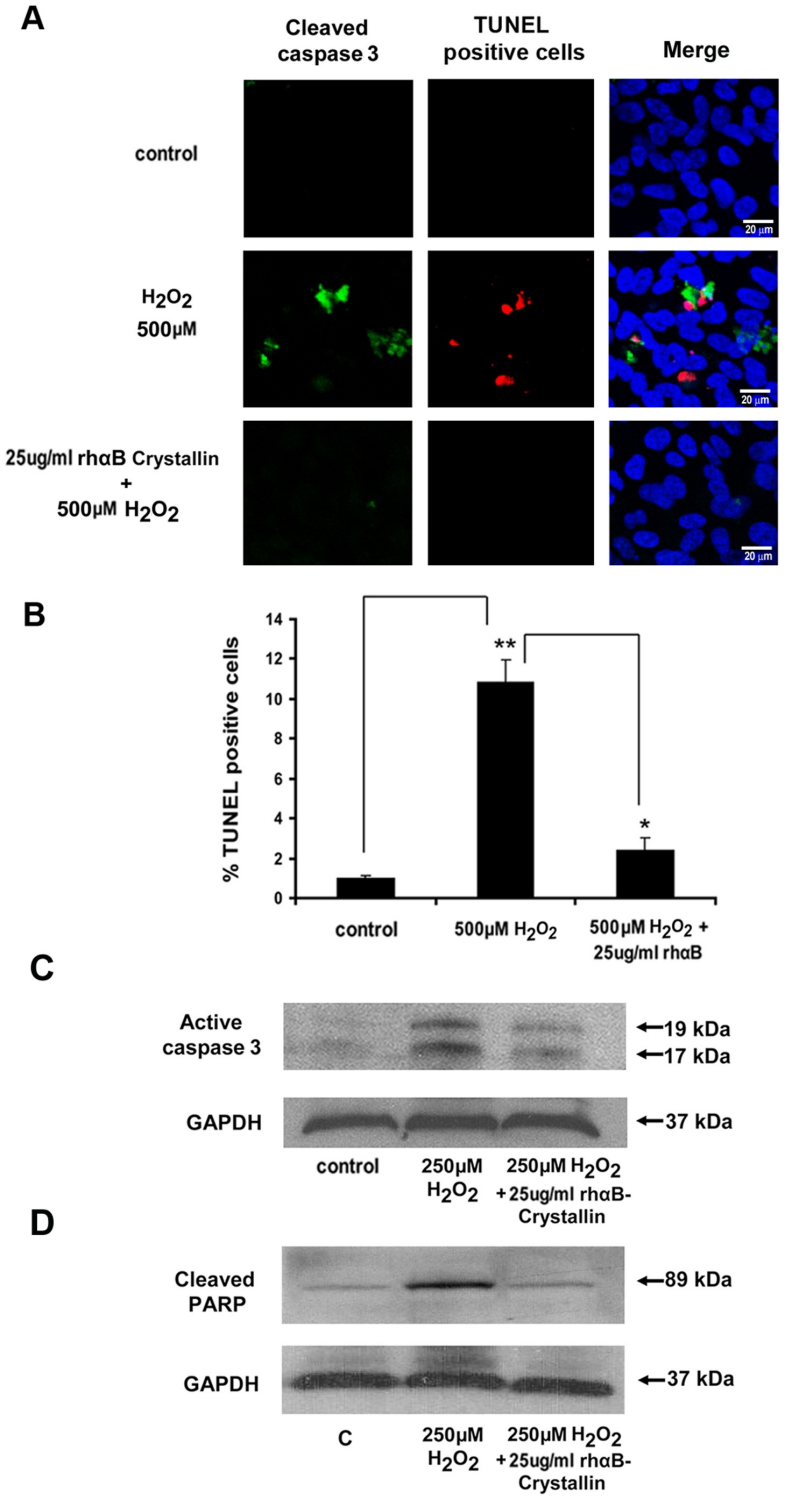
Exogenously added rhαB crystallin protects RPE cells from apoptosis. (A) Human RPE cells were treated with 500 µM H_2_O_2_ or 500 µM H_2_O_2_ plus 25 µg/ml rhαB crystallin for 3 h, and apoptosis was assessed by TUNEL staining and cleaved caspase 3 staining. Apoptosis and caspase 3 staining were significantly higher in H_2_O_2_-only treated cells when compared with cells co-treated with rhαB crystallin and H_2_O_2_. (B) Protection of RPE from apoptosis by 25 µg/ml rhαB crystallin in RPE cells treated with 500 µM H_2_O_2_ for 3 h. Quantification of the TUNEL-positive cells is shown. (C,D) Exogenously added rhαB crystallin protects RPE cells by inhibiting caspase 3 and PARP activation. RPE cells were treated with 250 µM H_2_O_2_ or 250 µM H_2_O_2_ and 25 µg/ml rhαB crystallin for 24 h. Caspase 3 activation (C) and PARP activation (D) was inhibited with rhαB crystallin co-treatment. red: TUNEL positive cells, green: cleaved caspase 3, blue: nuclear marker. Scale bar = 20 µm. *p<0.05, ** *p*<0.01.

Pronounced secretion of αB crystallin to the apical photoreceptor facing side of the RPE raised a strong possibility that αB crystallin may serve to protect adjacent cells, including photoreceptors and RPE cells. Therefore, the extracellular distribution of αB crystallin in the murine neural retina, especially in the IPM, was examined. Consistent with a protective role, αB crystallin was found in the IPM, where it co-localized with IRBP, an IPM marker (Figure 6A). Immunogold labeling in mouse retinas with αB crystallin antibody showed clear evidence for localization of αB crystallin in the IPM and photoreceptors (Figure 6B). To test the hypothesis that extracellular αB crystallin is internalized into neighboring cells, RPE cells were incubated with labeled rhαB crystallin in the presence or absence of mild and severe oxidative stress. Oxidative stress from H_2_O_2_ (150 µM and 500 µM for 1 h) caused uptake of the rh αB crystallin by the cytosol and nucleus. Uptake was greater with 500 µM H_2_O_2_ while it was negligible in control cells (Figure 6C). In separate experiments, we also investigated the effect of exogenous αB crystallin in mouse retinal explants exposed to 500 µM H_2_O_2_ for 30 min. Co-incubation with 25 µg/ml rhαB crystallin caused a significant uptake of αB crystallin by the outer and inner segments of photoreceptors under stressed conditions (Figure 6D). To examine whether the extracellular αB crystallin has any protective role, RPE cells were co-treated with 500 µM H_2_O_2_ and 25 µg/ml rhαB crystallin for 3 h. TUNEL and cleaved caspase 3 staining revealed a significant (*p*<0.01) suppression of apoptosis and cleaved caspase 3 expression by exogenous αB crystallin under stressed conditions (Figures 7A, B). Prolonged co-treatment of RPE cells with H_2_O_2_ (250 µM, 24h) and 25 µg/ml rhαB crystallin inhibited caspase 3 and PARP activation (Figure 7 C, D).

## Discussion

Our data provide strong evidence that RPE cells secrete αB crystallin. The secretion did not involve the classical pathway but was through exosomes and was lipid raft dependent. We also provide evidence that polarized human RPE monolayers secrete αB crystallin preferentially to the photoreceptor facing apical side. Oxidative stress caused disruption of tight junctions and was associated with accumulation of αB crystallin in the medium on the choroidal side as well. *In vivo*, localization of αB crystallin in IPM was observed. We hypothesized that αB crystallin secreted from RPE cells towards the photoreceptors could provide a protective environment and that, upon stress, it may internalize to mitochondria, endoplasmic reticulum, and nucleus to initiate antiapoptotic events. Consistent with this hypothesis, oxidative stress resulted in increased cellular uptake and accumulation of αB crystallin in the cytosol and nucleus by RPE cells. Finally, our data showed that exogenous αB crystallin protected RPE cells from oxidant-induced cell death and uptake by outer and inner segments of photoreceptors in stressed retinal explants.

Heat shock proteins are essential intracellular chaperones [26]; several family members of these proteins, normally localized to the cytosol, nucleus, or mitochondria, are released to the extracellular medium where they function as intercellular signaling ligands [24], [27]. A proteomic study conducted with oxidatively induced basolateral blebs in the ARPE-19 cell line revealed a wide variety of proteins, including αB crystallin [28]. In the present study, we characterized and quantified secretion of αB crystallin from early passage human RPE cells. It is noteworthy that the secretion occurs despite αB crystallin lacking a secretory sequence. αB crystallin resembles HSP70 [29] and basic fibroblast growth factor [30] in this respect. The amount of secretion in 24 h from RPE cells is only a small fraction of the steady state levels found in cytosolic and mitochondrial compartments. However, polarization of the RPE monolayer increased αB crystallin secretion five-fold when compared with nonpolarized RPE cells. A similar polarity-dependent secretion pattern was recently reported by our laboratory for pigment epithelial derived factor [19].

Our work has also demonstrated a novel secretory pathway for αB crystallin release that may be relevant to all types of cells. We found that the release of αB crystallin occurs by a mechanism other than the common secretory pathway [21], [22]. This conclusion was based on the finding that BF, an inhibitor of protein transport through the endoplasmic reticulum-Golgi complex, or TM, a glycosylation inhibitor, did not significantly inhibit αB crystallin release. Thus, although these drugs significantly interfere with the classical exocytic pathway, they did not affect αB crystallin release, demonstrating a pathway independent of the classical secretory route. However, our study supports the involvement of lipid rafts in αB crystallin release. Lipid rafts are thought to be involved in exosome formation [31]. After treatment with a lipid raft-disrupting agent, αB crystallin release was significantly decreased, suggesting that the stability and integrity of lipid rafts is required for efficient extracellular release.

The large heterogeneity of retinal diseases leading to photoreceptor death and consequent loss of vision in patients requires development of novel therapeutic strategies. A promising approach has been to target dying photoreceptors with molecules known to act as neuroprotectants and, in such manner, to prevent disease or to delay its progression. RPE cells are likely to support photoreceptors by either cell-to-cell-mediated paracrine signaling or by secretion of factors into the intercellular matrix between RPE and photoreceptors, which consist of a complex matrix of proteins and polysaccharides and may serve as a repository or depot of neuroprotective molecules. αB crystallin has been reported to play a significant role in protecting retinal tissues during the destructive inflammation that occurs during bacterial endophthalmitis [32]. In αB crystallin knockout (KO) mice, bacterial infection resulted in increased levels of apoptosis with significant reduction in retinal function [32]. Our laboratory also reported similar findings with cobalt chloride-induced retinal degeneration, in which αB crystallin KO mice were highly susceptible to apoptosis [14]. We postulated that since systemic administration of αB crystallin is protective in inflammatory models of experimental autoimmune encephalomyelitis [33], crystallin secreted from RPE cells towards the photoreceptors or IPM could provide a protective environment and that, upon stress, the αB crystallin may internalize to cellular compartments. Although we demonstrated the apical secretion of αB crystallin by an exosomal pathway *in vitro*, αB crystallin was identified without apparent exosomes in the murine IPM by TEM; this suggests that αB crystallin may accumulate in the IPM *in vivo* without retention of exosomal membranes. αB Crystallin may, upon internalization into adjacent RPE or photoreceptors, activate transcription factors involved in cell survival or may interact with proteins in nuclei to promote refolding or target damaged proteins [34]. Consistent with the hypothesis, we found internalization of exogenous αB crystallin and cellular protection under conditions of oxidative stress using RPE cells and retinal explants. Further, inhibition of cleavage of caspase 3 and PARP suggests that αB crystallin is able to suppress the effector steps of apoptotic cell death in RPE. It is of interest that in human lens epithelial cells overexpressing αB crystallin when subjected to oxidative stress, caspase 3 activity and PARP activation were significantly reduced thereby preserving the integrity of mitochondria and arresting subsequent release of cytochrome c [35].

As mentioned above, exogenous systemic administration of human recombinant αB crystallin significantly reduced apoptotic cells by inhibiting caspase 3 activation in experimental autoimmune encephalomyelitis [33]. Our present work in mouse retinal explants exposed to H_2_O_2_ showed that exogenous αB crystallin was taken up by the inner and outer segments of photoreceptors. The precise mechanism of this uptake *in vivo* remains to be delineated. It may involve the process of molecular diffusion as has been characterized recently for lens capsule for γD crystallin [36].

αB Crystallin could also act in the same way as pigment epithelium-derived factor, which functions to maintain a highly differentiated state and survival of photoreceptors [37]. Our data support the contention that oxidative stress results in cellular uptake and accumulation of full-length crystallin in the cytosol and nucleus, where it could act as a nuclear chaperone. To support our findings, colocalization of αB crystallin with the splicing factor SC35 in the nucleus has been reported, suggesting a role for αB crystallin in splicing or in protection of the splicing machinery [38]. We conclude that αB crystallin synthesized by the RPE cells is secreted preferentially from the apical surface and is distributed apically to the RPE bordering the outer segments of photoreceptors.

Drusen contain a mixture of extracellular, intracellular, and blood proteins (39). It is unclear how intracellular proteins eventually accumulate in drusen. It has been reported that drusen contain αB crystallin [15], [39], [40], and our present study may offer a mechanism by which αB crystallin accumulates in drusen. Under severe conditions of oxidative stress, there is a significant decrease in tight junction proteins due to barrier breakdown of RPE monolayers. This breakdown, along with mitochondrial ROS, could trigger the release of αB crystallin-containing exosomes to the basolateral side. In support of this finding, recent studies have demonstrated co-localization of αB crystallin with CD63 in human AMD samples and increased expression of exosome markers surrounding Bruch's membrane in the old mouse eye [25]. αB Crystallin could accumulate in the sub-RPE space and eventually appear as deposits in the drusen. Our study also revealed that the apical secretion of αB crystallin is still maintained in oxidatively stressed RPE. Consistent with this finding, several reports demonstrate increased expression of αB crystallin in the outer neural retina under different pathological conditions associated with oxidative stress. For example, αB crystallin showed increased expression in rod outer segments and RPE following intense light exposure of rat retina [41]. In retinal tissue sections from patients with AMD, increased αB crystallin expression was found in photoreceptors in association with drusen [42], and in the outer nuclear layer in association with choroidal neovascular membranes [15]. In agreement with these immunohistochemical studies, a proteomic study using neural retinal samples with progressive stages of AMD demonstrated increased αB crystallin levels in late stages of AMD [43]. However, it remains to be determined whether the αB crystallin found in the photoreceptors in these pathologic retinas is a result of uptake from exosomes released from RPE into the IPM, or is locally produced by the photoreceptor cells. In addition, it is conceivable that exosomes released by RPE may contain other components such as miRNA that could influence behavior of adjacent RPE and photoreceptors [44].

In conclusion, αB crystallin is an antiapoptotic protein that is released from RPE cells via exosomes. Polarization of the RPE resulted in assymetrical release of this molecule to the neural retina, where it could provide neuroprotection for the light-sensitive photoreceptors. Severe oxidative stress caused significant breaks in tight junctions and release of a small fraction of αB crystallin to the choroidal side, which may account for its accumulation in drusen. Because of the known pleiotropic biological functions of exosomes, including immune response, antigen presentation, intracellular communication, and the transfer of RNA and proteins [45], further understanding of exosomal transport of growth factors and or antiapoptotic molecules in the retina in pathological conditions could provide valuable information for therapeutic strategies in ocular diseases.

## Supporting Information

Figure S1 Validation of an ELISA method for quantification of αB crystallin. αB crystallin levels from cytosol and mitochondria isolated from confluent human RPE cells are presented in the inset along with the amount secreted into the medium. Data are mean ± SD from 3 experiments.
(0.57 MB TIF)

Figure S2 Extracellular release of LDH from human RPE is unaffected by treatment with several inhibitors of protein transport. RPE cells were pretreated for 2 h with indicated doses of inhibitors after which LDH release in a 24 h period was measured. No significant change in LDH release was noticed with any of the treatments as compared to untreated controls. BF, brefeldin, TM, tunicamycin, CD, β-methyl cyclodextrin, DMA, dimethyl amiloride. Data are mean ± SD from 3 experiments.
(2.67 MB TIF)

Figure S3 Characterization of polarized RPE monolayers from long-term cultures of RPE cells. Cells were grown in transwell filters for a month and showed high resistance (386±86 Ω.cm2 ). Expression of the tight junction proteins ZO1 (A) and occludin (B) and the apical membrane marker Na/K ATPase (C) was characterized by confocal microscopy. Fig. C also includes the Z-stack image of Na/K ATPase (red) verifying that this protein is localized apically.
(1.24 MB TIF)

## References

[1] de Jong PT (2006). Age-related macular degeneration.. N Engl J Med.

[2] Ambati J, Ambati BK, Yoo SH, Lanchulev S, Adamis AP (2003). Age-related macular degeneration: etiology, pathogenesis, and therapeutic strategies.. Surv Ophthalmol.

[3] Röhlich P (1970). The interphotoreceptor matrix: electron microscopic and histochemical observations on the vertebrate retina.. Exp Eye Res.

[4] Adler AJ (1989). Selective presence of acid hydrolases in the interphotoreceptor matrix.. Exp Eye Res.

[5] Zarbin MA (2004). Current concepts in the pathogenesis of age-related macular degeneration.. Arch Ophthalmol.

[6] Holz FG, Pauleikhoff D, Klein R, Bird AC (2004). Pathogenesis of lesions in late age-related macular disease.. Am J Ophthalmol.

[7] Gething MJ, Sambrook J (1992). Protein folding in the cell.. Nature.

[8] Schmitt E, Gehrmann M, Brunet M, Multhoff G, Garrido C (2007). Intracellular and extracellular functions of heat shock proteins: repercussions in cancer therapy.. J Leukoc Biol.

[9] Nickel W (2003). The mystery of nonclassical protein secretion. A current view on cargo proteins and potential export routes.. Eur J Biochem.

[10] Horwitz J (1992). Alpha-crystallin can function as a molecular chaperone.. Proc Natl Acad Sci USA.

[11] Kamradt MC, Chen F, Cryns VL (2001). The small heat shock protein alpha B-crystallin negatively regulates cytochrome c- and caspase-8-dependent activation of caspase-3 by inhibiting its autoproteolytic maturation.. J Biol Chem.

[12] Yaung J, Jin M, Barron E, Spee C, Wawrousek EF (2007). Alpha-Crystallin distribution in retinal pigment epithelium and effect of gene knockouts on sensitivity to oxidative stress.. Mol Vis.

[13] Jin JK, Whittaker R, Glassy MS, Barlow SB, Gottlieb RA (2008). Localization of phosphorylated alphaB-crystallin to heart mitochondria during ischemia-reperfusion.. Am J Physiol Heart Circ Physiol.

[14] Yaung J, Kannan R, Wawrousek EF, Spee C, Sreekumar PG (2008). Exacerbation of retinal degeneration in the absence of alpha crystallins in an in vivo model of chemically induced hypoxia.. Exp Eye Res.

[15] De S, Rabin DM, Salero E, Lederman PL, Temple S (2007). Human retinal pigment epithelium cell changes and expression of alphaB-crystallin: a biomarker for retinal pigment epithelium cell change in age-related macular degeneration.. Arch Ophthalmol.

[16] Steiner-Champliaud MF, Sahel J, Hicks D (2006). Retinoschisin forms a mutli-molecular matrix and cytoplasmic proteins: interactions with beta2 laminin and alphaB-crystallin.. Mol Vis.

[17] Hauck SM, Schoeffmann S, Deeg CA, Gloeckner CJ, Swiatek-de Lange M (2005). Proteomic analysis of the porcine interphotoreceptor matrix.. Proteomics.

[18] Sonoda S, Spee C, Barron E, Ryan SJ, Kannan R (2009). A protocol for the culture and differentiation of highly polarized human retinal pigment epithelial cells.. Nat Protoc.

[19] Sonoda S, Sreekumar PG, Kase S, Spee C, Ryan SJ (2010). Attainment of polarity promotes growth factor secretion by retinal pigment epithelial cells: relevance to age-related macular degeneration.. Aging.

[20] Sreekumar PG, Zhou J, Sohn J, Spee C, Ryan SJ (2008). N-(4-hydroxyphenyl) retinamide augments laser-induced choroidal neovascularization in mice.. Invest Ophthalmol Vis Sci.

[21] Lancaster GI, Febbraio MA (2005). Exosome-dependent trafficking of HSP70: a novel secretory pathway for cellular stress proteins.. J Biol Chem.

[22] Broquet AH, Thomas G, Masliah J, Trugnan G, Bachelet M (2003). Expression of the molecular chaperone Hsp70 in detergent-resistant microdomains correlates with its membrane delivery and release.. J Biol Chem.

[23] Théry C, Amigorena S, Raposo G, Clayton A (2006). Isolation and characterization of exosomes from cell culture supernatants and biological fluids.. *Curr Protoc* Cell Biol Chapter.

[24] Gupta S, Knowlton AA (2007). HSP60 trafficking in adult cardiac myocytes: role of the exosomal pathway.. Am J Physiol Heart Circ Physiol.

[25] Wang AL, Lukas TJ, Yuan M, Du N, Tso MO (2009). Autophagy and exosomes in the aged retinal pigment epithelium: possible relevance to drusen formation and age-related macular degeneration.. PLoS One.

[26] Glover JR, Lindquist S (1998). Hsp104, Hsp70, and Hsp40: a novel chaperone system that rescues previously aggregated proteins.. Cell.

[27] Asea A, Kabingu E, Stevenson MA, Calderwood SK (2000). HSP70 peptide-bearing and peptide-negative preparations act as chaperokines.. Cell Stress Chaperones.

[28] Alcazar O, Hawkridge AM, Collier TS, Cousins SW, Bhattacharya SK (2009). Proteomics characterization of cell membrane blebs in human retinal pigment epithelium cells.. Mol Cell Proteomics.

[29] Hightower LE, Guidon PT (1989). Selective release from cultured mammalian cells of heat-shock (stress) proteins that resemble glia-axon transfer proteins.. J Cell Physiol.

[30] Mignati P, Morimoto T, Rifkin DB (1992). Basic fibroblast growth factor, a protein devoid of secretory signal sequence, is released by cells via a pathway independent of the endoplasmic reticulum-Golgi complex.. J Cell Physiol.

[31] de Gassart A, Geminard C, Fevrier B, Raposo G, Vidal M (2003). Lipid raft-associated protein sorting in exosomes.. Blood.

[32] Whiston EA, Sugi N, Kamradt MC, Sack C, Heimer SR (2008). AlphaB-crystallin protects retinal tissue during Staphylococcus aureus-induced endophthalmitis.. Infect Immun.

[33] Ousman SS, Tomooka BH, van Noort JM, Wawrousek EF, O'Connor KC (2007). Protective and therapeutic role for alphaB-crystallin in autoimmune demyelination.. Nature.

[34] Bryantsev AL, Chechenova MB, Shelden EA (2007). Recruitment of phosphorylated small heat shock protein Hsp27 to nuclear speckles without stress.. Exp Cell Res.

[35] Mao YW, Liu JP, Xiang H, Li DW (2004). Human alphaA- and alphaB-crystallins bind to Bax and Bcl-X(S) to sequester their translocation during staurosporine-induced apoptosis.. Cell Death Differ.

[36] Danysh BP, Patel TP, Czymmek KJ, Edwards DA, Wang L, Pande J (2010). Characterizing molecular diffusion in the lens capsule.. Matrix Biol.

[37] Karakousis PC, John SK, Behling KC, Surace EM, Smith JE (2001). Localization of pigment epithelium derived factor (PEDF) in developing and adult human ocular tissues.. Mol Vis.

[38] van Rijk AE, Stege GJ, Bennink EJ, May A, Bloemendal H (2003). Nuclear staining for the small heat shock protein alphaB-crystallin colocalizes with splicing factor SC35.. Eur J Cell Biol.

[39] Crabb JW, Miyagi M, Gu X, Shadrach K, West KA (2002). Drusen proteome analysis: an approach to the etiology of age-related macular degeneration.. Proc Natl Acad Sci USA.

[40] Nakata K, Crabb JW, Hollyfield JG (2005). Crystallin distribution in Bruch's membrane-choroid complex from AMD and age-matched donor eyes.. Exp Eye Res.

[41] Sakaguchi H, Miyagi M, Darrow RM, Crabb JS, Hollyfield JG (2003). Intense light exposure changes the crystallin content in retina.. Exp Eye Res.

[42] Johnson PT, Brown MN, Pulliam BC, Anderson DH, Johnson LV Synaptic pathology, altered gene expression, and degeneration in photoreceptors impacted by drusen (2005).. Invest Ophthalmol Vis Sci.

[43] Ethen CM, Reilly C, Feng X, Olsen TW, Ferrington DA (2006). The proteome of central and peripheral retina with progression of age-related macular degeneration.. Invest Ophthalmol Vis Sci.

[44] Yuan A, Farber EL, Rapoport AL, Tejada D, Deniskin R (2009). Transfer of microRNAs by embryonic stem cell microvesicles.. PLoS One.

[45] Simpson RJ, Lim JW, Moritz RL, Mathivanan S (2009). Exosomes: proteomic insights and diagnostic potential.. Expert Rev Proteomics.

