# Supplementary figures and images for: αB Crystallin Is Apically Secreted within Exosomes by Polarized Human Retinal Pigment Epithelium and Provides Neuroprotection to Adjacent Cells
Source: PLoS One. 2010 Oct 8;5(10):e12578. doi: 10.1371/journal.pone.0012578 (PMC2951891; doi:10.1371/journal.pone.0012578)

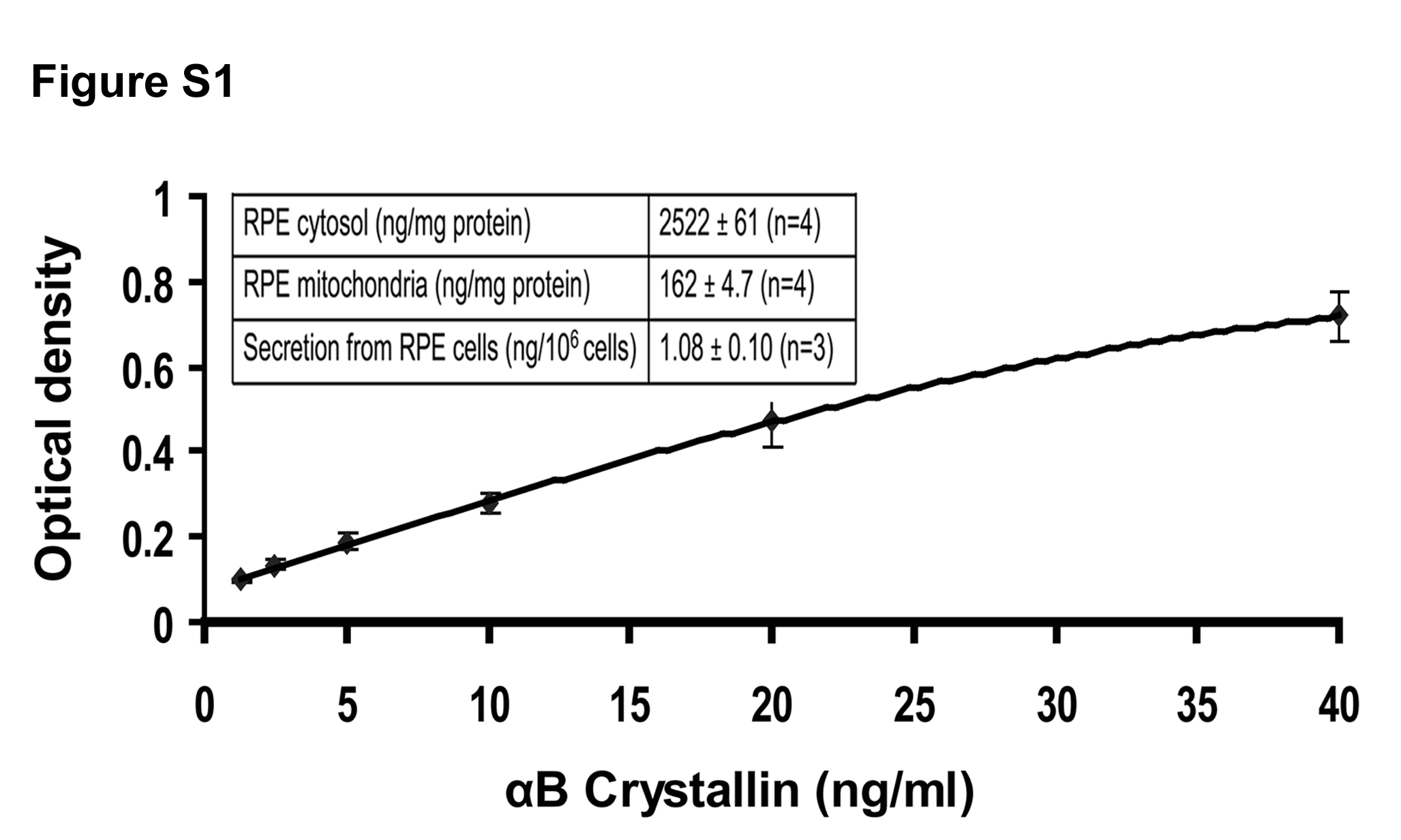

Supplement: Figure S1 — Validation of an ELISA method for quantification of αB crystallin. αB crystallin levels from cytosol and mitochondria isolated from confluent human RPE cells are presented in the inset along with the amount secreted into the medium. Data are mean ± SD from 3 experiments. (0.57 MB TIF) [file pone.0012578.s001.tif]

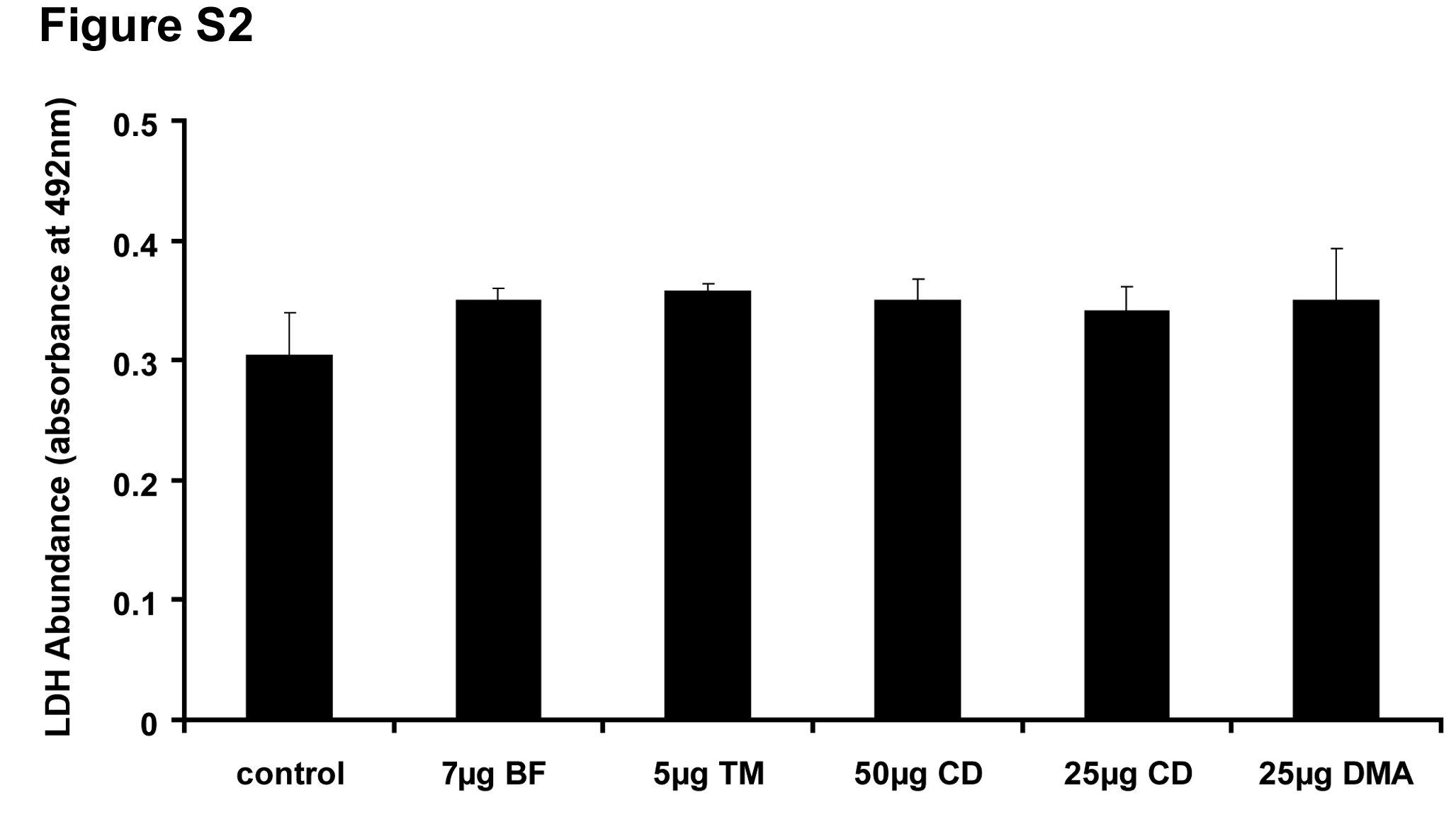

Supplement: Figure S2 — Extracellular release of LDH from human RPE is unaffected by treatment with several inhibitors of protein transport. RPE cells were pretreated for 2 h with indicated doses of inhibitors after which LDH release in a 24 h period was measured. No significant change in LDH release was noticed with any of the treatments as compared to untreated controls. BF, brefeldin, TM, tunicamycin, CD, β-methyl cyclodextrin, DMA, dimethyl amiloride. Data are mean ± SD from 3 experiments. (2.67 MB TIF) [file pone.0012578.s002.tif]

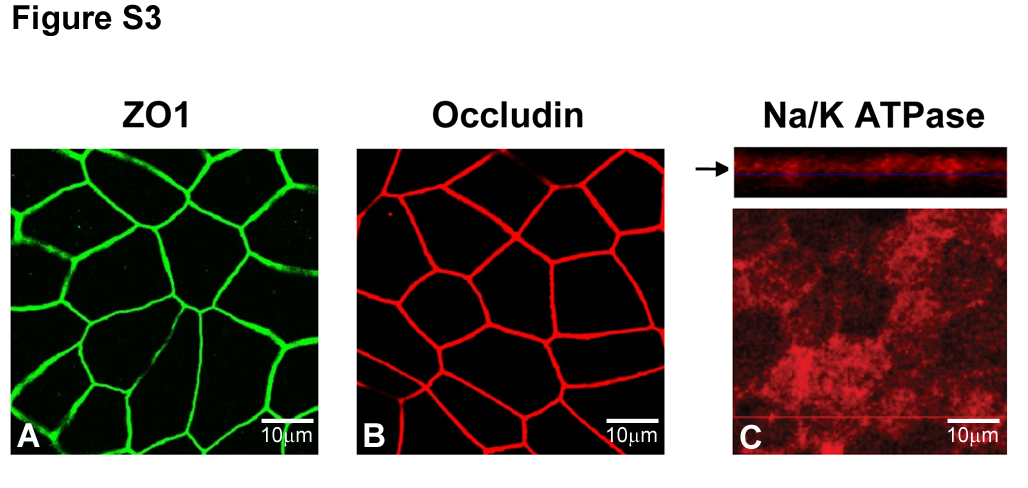

Supplement: Figure S3 — Characterization of polarized RPE monolayers from long-term cultures of RPE cells. Cells were grown in transwell filters for a month and showed high resistance (386±86 Ω.cm2 ). Expression of the tight junction proteins ZO1 (A) and occludin (B) and the apical membrane marker Na/K ATPase (C) was characterized by confocal microscopy. Fig. C also includes the Z-stack image of Na/K ATPase (red) verifying that this protein is localized apically. (1.24 MB TIF) [file pone.0012578.s003.tif]
